# Molecular Mechanisms of Colistin Resistance in *Klebsiella pneumoniae* in a Tertiary Care Teaching Hospital

**DOI:** 10.3389/fcimb.2021.673503

**Published:** 2021-10-26

**Authors:** Yanling Liu, Yiqing Lin, Ziwen Wang, Niya Hu, Qiong Liu, Wenkai Zhou, Xiuzhen Li, Longhua Hu, Jian Guo, Xiaotian Huang, Lingbing Zeng

**Affiliations:** ^1^ Department of Clinical Laboratory, The First Affiliated Hospital of Nanchang University, Nanchang, China; ^2^ Department of Medical Microbiology, School of Medicine, Nanchang University, Nanchang, China; ^3^ Department of Clinical Laboratory, The Second Affiliated Hospital of Nanchang University, Nanchang, China; ^4^ Department of Laboratory Medicine, Shanghai East Hospital, Tongji University School of Medicine, Shanghai, China

**Keywords:** *Klebsiella pneumoniae*, Colistin resistance, mcr, Pan-drug resistance, Molecular mechanism

## Abstract

**Background:**

Over the last two decades, the prevalence of colistin resistance among the members of *Enterobacteriaceae* has been increasing, particularly among *Klebsiella pneumoniae* isolates; this limits the potential use of colistin and leads to worsened clinical outcomes.

**Methods:**

We investigated the prevalence and genetic characteristics of colistin-resistant *K. pneumoniae* (COLR-KP) in clinical isolates using genomic sequencing.

**Results:**

In total, 53 K*. pneumoniae* isolates (4.5%, 53/1,171) were confirmed as COLR-KP, of which eight isolates carried mobile colistin-resistant (*mcr*) gene. Although the overall prevalence rate (0.7%, 8/1,171) of *mcr*-like genes in clinical *K. pneumoniae* remained relatively low, the presence of *mcr* (15.1%, 8/53) among the COLR-KP isolates indicated that the mobile resistance gene was already widespread among *K. pneumoniae* isolates in hospital setting. We randomly selected 13 COLR-KP isolates (four *mcr*-bearing and nine non-*mcr*-bearing isolates) for whole-genome sequencing, including two pandrug-resistant and four sequence type 11 (ST11) isolates. Phylogenetic analysis revealed that all COLR-KP isolates were genetically diverse. Among the four *mcr*-bearing isolates, three (KP4, KP18, and KP30) were positive for *mcr-1* and one (KP23) for *mcr-8*; none of the other *mcr* genes were detected. The *mcr-1* in the KP4 and KP30 isolates were located in an *IncX4* plasmid (approximately 33 kb) and could be successfully transferred to *Escherichia coli* J53AZ^R^. In contrast, for the *mcr-8*-bearing plasmid in KP23 (*IncFII*), colistin resistance could not be transferred by conjugation. The mcr-1-producing isolate KP18 coexists a novel plasmid-carried tigecycline resistance gene tmexCD1-toprJ1. The most common chromosomal mutation associated with colistin resistance was a T246A amino acid substitution in PmrB, which was identified in most COLR-KP isolates (11/13, 84.6%). All ST11 isolates additionally had an R256G amino acid substitution. Critical virulence factors associated with hypervirulent *K. pneumoniae* were detected in four COLR-KP isolates; these virulence factors included aerobactin, salmochelin, and yersiniabactin.

**Conclusion:**

We found that *mcr*-bearing COLR-KP emerged in our hospital and was growing at an increasing rate. Simultaneous emergence of hypervirulence and colistin–tigecycline–carbapenem resistance in the epidemic clone ST11 *K. pneumoniae* was also observed; this highlights the significance of active and continuous surveillance.

## Background

Multidrug-resistant (MDR) gram-negative bacteria present a serious threat to global public health, especially, carbapenem-resistant *Klebsiella pneumoniae* (CRKP). Colistin has been considered one of the last resorts in treating severe infections that retain activity against *K. pneumoniae* carbapenemase (KPC)-producing *K. pneumoniae*. However, the emergence and global dissemination of colistin resistance compromise the efficacy of colistin.

Colistin is a cationic peptide synthesized by Gram-positive species, and it exhibits antimicrobial activity through multiple pathways, including direct antibacterial activity, antiendotoxin colistin activity, vesicle–vesicle contact pathway, hydroxyl radical death pathway, and respiratory enzyme inhibition (Sun et al., 2018). With antimicrobials, bacteria develop a series of resistance systems, including chromosomal gene mutations and plasmid-associated resistance genes to adapt to the environment. Colistin resistance in *K. pneumoniae* is mediated by several mechanisms. Mutations in genes encoding PhoP/PhoQ and PmrA/PmrB two-component regulatory systems regulate the expression of *pmrC* and *pmrHFIJKLM* operons and are responsible for the synthesis and transfer of 4-amino-4-deoxy-L-arabinose (L-Ara4N) cationic groups to lipid A. This modification increases the positive charge on lipopolysaccharides (LPSs) and therefore, decreases colistin binding, leading to colistin resistance (Poirel et al., 2017). Alternatively, MgrB is a small transmembrane protein produced upon the activation of the PhoP/PhoQ signaling system and acts as a negative regulator of this system. It regulates the expression of *etpB*, which is related to the modification of LPS. Mutations in *mgrB* gene upregulate the expression of the PhoP/PhoQ system, thereby leading to colistin resistance. Generally, *mgrB* mutations are caused by insertion sequences (ISs; IS*5-like*, IS*1F*, IS*Kpn13*, IS*Kpn14*, and IS*10R*) or point mutations (Cannatelli et al., 2014). In addition, hyperproduction of capsular polysaccharide reduces the interactions of colistin with the bacterial surface, leading to colistin resistance (Poirel et al., 2017). Besides, horizontal transfer of plasmid-borne *mcr* gene play a significant role in the dissemination of colistin resistance among various bacteria. Since its first identification in the late 2015 (Liu et al., 2016), the determinants of transferable colistin resistance have extended further away from *mcr-*1 to include various novel alleles (Sun et al., 2018). Until now, nine novel *mcr* variants have been reported, namely, *mcr-2*, *mcr-3*, *mcr-4*, *mcr-5*, *mcr-6*, *mcr-7*, *mcr-8*, *mcr-9*, and the recently identified *mcr-10* (Wang et al., 2018; Xiang et al., 2018; Yang et al., 2018; Kieffer et al., 2019; Wang et al., 2020). Moreover, the *mcr* family has been increasingly reported in various genera of *Enterobacteriaceae* worldwide, isolated from food animals, meat and vegetables, the environment, infected patients, and asymptomatic human carriers (Skov and Monnet, 2016). All MCR proteins are characterized as phosphoethanolamine (PEtN) transferases. They catalyze the attachment of PEtN to lipid A and lead to a reduction of the negative charge of LPS through structural alterations of lipid A and a decrease in the binding of colistin, thus resulting in colistin resistance (Sun et al., 2018). However, although these alleles belong to the group of PEtN transferases, the similarities in amino acid sequences vary, reflecting different genetic origins of these *mcr* alleles. Similarly, previous studies have demonstrated the complex dissemination of these alleles across the diversified species of *Enterobacteriaceae* and plasmid reservoirs and genetic environment for *mcr*-like genes (Wang Q. et al., 2017; El-Sayed Ahmed et al., 2020). Nevertheless, limited reports have indicated the presence of *mcr-1*, *mcr-3*, *mcr-7*, and *mcr-8* in *K. pneumoniae* at a relatively low prevalence. Although several studies have elucidated colistin-resistance mechanisms from different aspects, the underlying mechanism of colistin resistance in clinical *K. pneumoniae* isolates in our hospital was unclear.

Therefore, in this study, we identified 53 colistin-resistant *K. pneumoniae* (COLR-KP) isolates between 2017 and 2019 to investigate the primary mechanisms of colistin resistance and evaluate the potential prevalence of clinical *K. pneumoniae* isolates in our hospital.

## Material and Methods

### Bacterial Isolates and Antimicrobial Susceptibility Testing

In total, 1,171 *K. pneumoniae* clinical isolates and relevant data were collected between June 2017 and November 2019 at The First Affiliated Hospital of Nanchang University, a 3,200-bed tertiary care teaching hospital in Jiangxi, China. Matrix-assisted laser desorption ionization–time of flight mass spectrometry (BioMérieux, Marcy-l’Étoile, France) was used to identify the isolates. The ethics committee of the related university hospital (approval no. 2014036) approved this study. The VITEK 2 System (BioMérieux, Marcy-l’Etoile, France) was used to assess the *in vitro* antimicrobial susceptibility. The interpretation of tigecycline-related data was based on the criteria proposed by the US Food and Drug Administration. Similarly, the minimum inhibitory concentrations (MICs) of colistin were determined using the broth microdilution method in triplicate (Sigma, St. Louis, MO, USA) according to the Clinical and Laboratory Standards Institute guidelines (document VET01-A4) and interpreted following the European Committee on Antimicrobial Susceptibility Testing breakpoints (European Committee on Antimicrobial Susceptibility Testing. Breakpoint tables for the interpretation of MICs and zone diameters. EUCAST; 2019. Version 9.0. http://www.eucast.org/clinicalbreakpoints/). All colistin-non-susceptible isolates were defined as those with MIC >2 μg/ml.

### Plasmid-Mediated Colistin-Resistance Gene Screening and Conjugation Experiments

All COLR-KP isolates were screened for the presence of *mcr-1* to *mcr-8* genes (**Supplementary Table S1**) using polymerase chain reaction (PCR) and sequenced as described in the previous literature (Wang et al., 2018). The primers are listed in **Supplementary Table S1**.

Conjugation experiments were performed for *mcr* gene-bearing COLR-KP as described previously (Wang Y. et al., 2017). Briefly, using the azide-resistant *Escherichia coli* J53 as the recipient strain, both donor and recipient strains were cultured in the exponential phase and mixed on solid LB agar using filters at a 1:10 donor/recipient ratio. After 5 h of incubation, filters were resuspended in 0.9% NaCl, and the bacterial mixture was plated onto LB agar plates supplemented with colistin (1 μg/ml) and sodium azide (100 μg/ml). Confirmation of the susceptibility of all transconjugants to antibiotics was conducted using the antibiogram, followed by the amplification of the *mcr* gene using PCR.

### Whole-Genome Sequencing

Genomic DNA was extracted from overnight cultures of selected isolates using the QIAGEN DNeasy Kit (Qiagen Sciences, Germantown, MD, USA) following the protocol of the manufacturer and sent for whole-genome sequencing (WGS). Genomic libraries were prepared with an insert of approximately 350 bp using the TruSeq DNA PCR-Free Sample Preparation Kit (Illumina Inc., San Diego, CA, USA) following the instructions of the manufacturer and sequenced using the Illumina HiSeq platform using a 150-bp paired-end protocol (Annoroad Biotech Co., Beijing, China). Two of the isolates were extracted using the QIAGEN Large-Construct Kit (Qiagen Sciences, Germantown, MD, USA) and sequenced using the PacBio RS II System (Pacific Bioscience, Menlo Park, CA, USA) with a 10-kb size library and P6/C4 chemistry.

Raw reads were trimmed with Trimmomatic 0.30 to remove low-quality sequences and adapters. Genomes were *de novo* assembled using the SPAdes Genome Assembler v3.13.0 (https://github.com/ablab/spades) (Bankevich et al., 2012).

### Bioinformatics Analysis

General genomic features were defined by PATRIC automatic annotation tools. Mobile antibiotic resistance genes, including plasmid-mediated colistin-resistance genes, were identified using ResFinder 3.0 (https://cge.cbs.dtu.dk/services/ResFinder/) (Zankari et al., 2012). Plasmid replicon types were determined using PlasmidFinder v2.0 (https://cge.cbs.dtu.dk/services/PlasmidFinder/) with a minimum threshold of 95% identity (Carattoli et al., 2014). ISs were identified using ISfinder (Siguier et al., 2006). *In silico* multilocus sequence typing and serotyping were confirmed using MLST v.2.11 (https://cge.cbs.dtu.dk/services/MLST/) and Kaptive Web (https://github.com/katholt/Kaptive), respectively (Wick et al., 2018). Plasmid alignment was performed using the BRIG (http://brig.sourceforge.net/) software.

### Mutations in Colistin-Resistance Genes

Mutations in genes potentially involved in colistin resistance (*mgrB*, *pmrA/pmrB*, and *phoP/phoQ*) were inspected by alignment with reference genome *K. pneumoniae* subsp*. pneumoniae* MGH78578 (# NC_009648.1). The PROVEAN tool v.1.1.5 (http://provean.jcvi.org/index.php) was used to predict the effect of amino acid substitutions on protein function (Choi and Chan, 2015). PROVEAN score ≤−2.5 was deleterious for protein function, and a score >−2.5 was considered to have a neutral effect on protein function.

### Comparative Genomic and Phylogenetic Analysis

All selected genomes in this study and the reference genome *K. pneumoniae* subsp. *pneumoniae* MGH78578 were annotated with RAST (Overbeek et al., 2014). Genomes were aligned, and the core genome was inferred with Roary v.3.11.2 (Page et al., 2015). A maximum-likelihood phylogenetic tree was inferred by PhyML v.3.1 using the GTR evolutionary model with 500 bootstraps (Guindon et al., 2010). The phylogenetic tree was visualized using MEGA 6.0 (Kumar et al., 2018).

## Results

### General Characteristics of *K.pneumoniae* Isolates

During the study period, we collected 1,171 non-duplicate isolates from various clinical samples. A total of 53 *K. pneumoniae* isolates (4.5%, 53/1,171) were then confirmed as COLR-KP, of which eight carried plasmid-mediated colistin-resistant *mcr* genes. Despite the relatively low overall prevalence rate (0.7%, 8/1,171) of *mcr*-like genes in clinical *K. pneumoniae* isolates, the presence of *mcr* (15.1%, 8/53) among the COLR-KP isolates indicated that this gene was already widely disseminated among *K. pneumoniae* isolates in our hospital. Of the 53 COLR-KP isolates, 13 (four *mcr*-bearing and nine non-*mcr*-bearing isolates) were randomly selected for further characterization, with colistin MICs ranging from 8 to 128 µg/ml, as shown in **Table 1**.

**Table 1:** Clinical features, resistance profiles, and sequence types of colistin-resistant *Klebsiella pneumoniae* isolates.

Isolate	Date(year)	Specimen	CRO	TZP	MEM	IPM	GN	AK	LEV	ATM	TGC	COL	ST
KP3	2018	Urine	≥64	≥128	≥16	≥16	≥16	≤8	≥8	≥64	≤2	8	11
KP4	2018	Blood	≤1	≤16	≤1	≤1	≥16	≤16	≤2	≤4	≤2	8	25
KP7	2018	Urine	≥64	≤4	≤1	≤1	≥16	≤2	4	16	≤2	128	36
KP9	2018	Sputum	≥64	≥128	≤1	≤1	≤4	≤16	≥8	≥64	≤2	32	22
KP11	2018	Sputum	≤1	≤16	≤1	≤1	≤4	≤8	≤2	≤4	≤2	16	592
KP12	2018	Sputum	≤1	≤16	≤1	≤1	≤4	≤8	≤2	≤4	≤2	16	105
KP15	2018	Sputum	≥64	≥128	≥16	≥16	≥16	≥64	≥8	≥64	≤2	8	11
KP18	2018	Surgical wound	32	≥128	≤1	≤1	≥16	≥64	≥8	≥64	≤2	16	378
KP19	2018	Sputum	≤1	≤16	≤1	≤1	≤4	≤16	≤2	≤4	≤2	16	218
KP23	2018	Surgical wound	≥64	≥128	≤1	≤1	≥16	≤16	≥8	≥64	≤2	16	11
KP30	2018	Ascites	≥64	≤4	≤1	≤1	≤1	≤2	≤0.25	16	≤2	64	294
KP67	2019	Blood	≥64	≥128	≥16	≥16	≥16	≥64	≥8	≥64	8	64	11
KP69	2019	Blood	≥64	≥128	≥16	≥16	≥16	≥64	≥8	≥64	8	64	11

ST, sequence type; BSI, bloodstream infection; UTI, urinary tract infection; CRO, ceftriaxone; TZP, piperacillin–tazobactam; MEM, meropenem; IPM, imipenem; GN, gentamicin; AK, amikacin; LEV, levofloxacin; ATM, aztreonam; TGC, tigecycline; COL, colistin.

Among the 13 isolates, eight were isolated from sterile specimens, including blood, urine, peritoneal fluid, and surgical wound, whereas the other five were isolated from non-sterile sputum specimen. Patients were aged 35–80 (average 64) years. All patients had a history of previous hospitalization but without exposure to colistin, none had a history of recent overseas travel. Therefore, it was presumed that the spread of colistin resistance occurs in hospitals without colistin use. In the bloodstream isolates, designated KP4, KP67, and KP69, KP4 was recovered from a patient with severe bloodstream infection (BSI) with acute monocytic leukemia. This patient was under immunosuppression and already received combination treatments of imipenem, meropenem, teicoplanin, and voriconazole. Alternatively, patients expressing KP67 and KP69 were admitted for third-degree burn injuries, where the barrier of their skin mucosa was severely damaged.

Antimicrobial susceptibility testing revealed that of these COLR-KP isolates, four were assigned to CRKP and five were extended-spectrum beta-lactamase (ESBL)-producing isolates. Both exhibited MDR phenotypes, whereas the remaining five isolates were susceptible to most antimicrobials other than colistin. This finding was similar to previous observations of colistin resistance developed in MDR strains. This result thus indicates that colistin resistance evolved independently.

*In silico* MLST analysis assigned the COLR-KP isolates to nine distinct STs, of which five belonged to ST11 (5/13, 38.5%) and the rest belonged to different STs. Two of these ST11 isolates were recovered from the blood of patients with burn injury who displayed pan-resistance profiles and were resistant to all tested antimicrobial agents, including tigecycline. STs and antimicrobial-resistance profiles are shown in **Table 1**.

### General Genomic Features

A summary of the genomic features of the 13 sequenced *K. pneumoniae* genomes is presented in **Table S2**. Isolates KP67 and KP69 were two completed genomes, and the rest were draft sequences. Whole-genome sizes ranged from 5,284,652 to 5,958,900 bp, with an average GC content of 57.2%. The mean number of mapped contigs was 231. The coding sequences ranged from 3,521 to 6,100, and the proteins with assignment function ranged from 3,083 to 5,189.

### Antimicrobial Resistance and Virulence Genes

Four COLR-KP isolates (KP3, KP15, KP67, and KP69) were carbapenem-resistant, which belonged to ST11, and were positive for *bla*_KPC-2_ and ESBL gene *bla*_CTX-M-65_, corresponding to a previous national surveillance (Zhang et al., 2017). ESBL genes, mainly of the CTX-M group, including *bla*_CTX-M-3_, *bla*_CTX-M-14_, and *bla*_CTX-M-65_, were identified in six of the 13 COLR-KP isolates. Isolate KP18 bore tigecycline-resistance gene *tmexCD1-toprJ1*; however, the strain remained susceptible to tigecycline while being resistant to other antimicrobials such as tetracyclines, quinolones, and aminoglycosides. These COLR-KP isolates also harbored several antimicrobial-resistance genes leading to *β*-lactam, aminoglycoside, fluoroquinolone, sulfonamide, and trimethoprim resistance. Four isolates also possessed multiple virulence genes, including *rmpA*, *iucABCD*, *iroBCDN*, *iutA*, and *ybt*, which have been associated with urinary tract infections, septicemia, and pneumonia. KP4 and KP67 isolates were obtained from patients with BSI who finally died of severe infection. The antimicrobial-resistance genes, virulence genes, and serotypes of COLR-KP isolates are shown in **Table 2**.

**Table 2:** Antimicrobial-resistance genes, virulence genes, and serotypes of colistin-resistant *Klebsiella pneumoniae* isolates.

Isolate	Resistance genes					Virulent				Plasmids
	*β*-Lactam	Fluoroquinolone	Aminoglycoside	Tigecycline (acquired)	Others	Virulence genes	wzi	Cps	O locus	Predicted plasmid elements
KP3	*bla*_KPC-2_, *bla*_CTX-M-14_, *bla*_CTX-M-55_, *bla*_SHV-182_, *bla*_TEM-1B_	*qnrS1*	*aac(3)-IId,aadA2b, aph(3’’)-Ib,aph(6)-Id*		*fosA,sul1,sul2,tet(A),dfrA1,catA2*	*ybt 9*	64	K64	O2	IncFIB(K), IncFII, IncFII, IncR,ColRNAI
KP4	*bla*_SHV-110_, *bla*_SHV-81_	*-*	*aac(3)-IId,aph(3’’)-Ib,aph(3′)-Ia,aph(3′)-Id*		*sul1,sul2,dfrA1*	*iucABCD, iutA*	72	K2	O1	IncX4, IncFIA(HI1), IncFIB(K), IncFIB, IncFII, IncN
KP7	*bla*_CTX-M-3_, *bla*_SHV-11_, *bla*_SHV-13_, *bla*_SHV-70_, *bla*_TEM-1B_	*aac(6′)-Ib-cr,qnrB2,qnrB52,qnrS1,oqxA,oqxB*	*aac(3)-IId,aac(6′)-Ib-cr,aadA16,aph(3’’)-Ib,aph(3’)-Ia,aph(6)-Id*		*fosA5,floR,mph(A),ARR-3,sul1,sul2,tet(A),dfrA27*		27	K27	O2	IncFII(K)
KP9	*bla*_CTX-M-14_, *bla*_DHA-1_, *bla*_SHV-99_	*oqxA,oqxB,qnrB4*			*fosA,sul1*		9	K9	O1	IncFIA(HI1)
KP11	*bla*_SHV-26_	*oqxA, oqxB*			*fosA5*	*rmpA2, iucABCD, iutA, iroBCDN*	206	K57	O3b	IncHI1B
KP12	*bla*_DHA-1_,*bla*_SHV-187_	*oqxA, oqxB, qnrB4*	*aadA16*		*fosA,ARR-3, sul1, tet(B), dfrA27*	*ybt*	383	K102	O2	IncHI1B
KP15	*bla*_KPC-2_,*bla*_SHV-182_,*bla*_TEM-1B_	*oqxA, oqxB*	*aadA2b, aph(3’’)-Ib*, *aph(6)-Id, rmtB*		*fosA, fosA3, sul1, sul2, tet(A), dfrA1, catA2*	*ybt 9*	64	K64	O2	IncFIB(K), IncFII, IncFII, IncR, ColRNAI
KP18	*bla*_SHV-119_,*bla*_DHA-1_	*qnrB6, qnrB4, aac(6′)-Ib-cr, oqxA, oqxB*	*aph(3′)-Ia, aac(6′)-Ib-cr, aadA2, aadA16, aadA1, aph(6)-Id, aph(4)-Ia, armA, aac(3)-IV, aph(3’’)-Ib*	*tmexCD1-toprJ1*	*fosA, msr(E), mph(A), mph(E), ARR-3, sul1, sul2, sul3, tet(A), dfrA27*		177	K125	O5	IncFIA(HI1), IncFIB(K), IncFIB(Mar), IncFIB(pKPHS1), IncFII(K), IncHI1B, IncR
KP19	*bla*_SHV-33_	*oqxA, oqxB*			*fosA*	*rmpA2, iucABCD, iroBCDN, iutA, ybt 9*	77	K57	O2	
KP23	*bla*_CTX-M-3_, *bla*_SHV-182_, *bla*_TEM-1B_	*aac(6’)-Ib-cr, qnrS1*	*aac(3)-IId, aph(3’)-Ia, aac(6’)-Ib-cr, aph(3’’)-Ib, aph(6)-Id, aadA16*		*fosA, ARR-3, sul2, tet(A), dfrA27*		385	K111	O3b	IncFII(K), IncFIA(HI1), IncR
KP30	*bla*_SHV-187_	*oqxA,oqxB*			*fosA*	*ybt 4*	274	K30	O1	IncX4, IncHI1B
KP67	*bla*_KPC-2,_ *bla*_CTX-M-65_, *bla*_SHV-12_, *bla*_LAP-2_, *bla*_TEM-1D_	*qnrS1*	*rmtB*		*sul2, tet(A), dfrA14, CatA2*	*rmpA2, iucABCD, iutA,ybt 9*	64	K64	O2	IncHI1B, IncFII, IncR, colRNAI, repB
KP69	*bla*_KPC-2_, *bla*_CTX-M-65_, *bla*_SHV-11_, *bla*_TEM-1D_		*aadA2, rmtB, strA, strB*		*fosA3, sul1, sul2, tet(A), dfrA1, CatA2*	*ybt 9*	64	K64	02	IncFIB(K), IncFII(pKP91), IncR, colRNAI

### Colistin-Resistance Mechanisms

Four isolates were confirmed as *mcr*-bearing COLR-KP, of which three (KP4, KP18, and KP30) were positive for *mcr-1*and one (KP23) for *mcr-8*; none of the other *mcr* genes was detected. WGS and plasmid replicon typing revealed that *mcr-1.1* in KP4 and KP30 isolates was located in an *IncX4* plasmid (approximately 33 kb). The *mcr-1* bearing plasmid pMCR_KP18 in KP18 coexisted with an acquired novel tigecycline-resistance gene in an *IncHI1* plasmid. Conjugation experiments showed that colistin resistance could be successfully transferred from *mcr-1*-bearing isolates KP4, KP18, and KP30 to *E. coli* J53AZ^R^, with the colistin MIC for the transconjugants increasing 16- and 4-fold compared with the untransformed control, respectively. PCR and sequencing analysis further revealed that the transconjugants harbored *mcr-1* gene, which demonstrated that the *mcr-1* genes in isolates KP4, KP18, and KP30 were functional in colistin resistance.

The *mcr-8*-bearing plasmid in KP23 was identified as an *IncFII* type of approximately 13 kb in size. In contrast, colistin resistance from *mcr-8*-KP23 strain could not be transferred by conjugation despite several attempts. No genes encoding conjugation-related proteins were found in the complete sequence of pMCR_KP23. Therefore, it is suggested that the *mcr-8*-bearing plasmid in this strain was non-self-transmissible.

We also screened for common chromosomal mutations that caused resistance to colistin. All COLR-KP isolates had wild-type *phoP* and similar neutral amino acid substitution T246A in *pmrB* gene. In addition, five ST11 isolates coexisted with missense mutations in *pmrB* leading to R256G substitution. Isolate KP15 also had a missense mutation as a T157P substitution. Alterations in the PhoP/PhoQ regulator *mgrB* gene occurred in three isolates; KP12 and KP69 had complete deletion in *mgrB*, whereas the ST11 isolate KP67 had an interruption in the *mgrB* gene caused by a promoter deletion. These three isolates did not possess any *mcr* gene or other deleterious mutation. Therefore, the inactivation of *mgrB* gene might be responsible for its high colistin resistance (Hamel et al., 2020). Moreover, four *mcr*-bearing isolates had chromosomal mutations in the *pmrB*, leading to T246A substitution and absence of *phoP* mutations. KP23 isolate additionally had a deleterious R256G amino acid substitution in *pmrB* and KP 18 had two amino acid mutations in pmrA (M66I, E35A) (**Table 3**).

**Table 3:** Plasmid-mediated genes and chromosomal mutations related to colistin resistance in *Klebsiella pneumoniae* isolates.

Isolate	*mcr* gene	Chromosomal mutation*		
		*mgrB*	*pmrB*	Others
KP3			T246A, R256G	*phoQ*(V24G)
KP4	*mcr-1*		T246A	
KP7			T246A	
KP9			T246A, S203P	
KP11			T246A	
KP12		Complete deletion	T246A	
KP15			T246A, R256G, T157P	
KP18	*mcr-1*		T246A	*pmrA*(M66I,E35A)
KP19			T246A, V257A, T240M	
KP23	*mcr-8*		T246A, R256G	
KP30	*mcr-1*		T246A, M285L	
KP67		Promoter area deletion		
KP69		Complete deletion		

*Aligned with Klebsiella pneumoniae subsp. pneumoniae MGH 78578 (GenBank accession no. CP000647).

### Comparative Genomic Analysis of COLR-KP

**Figure 1:**
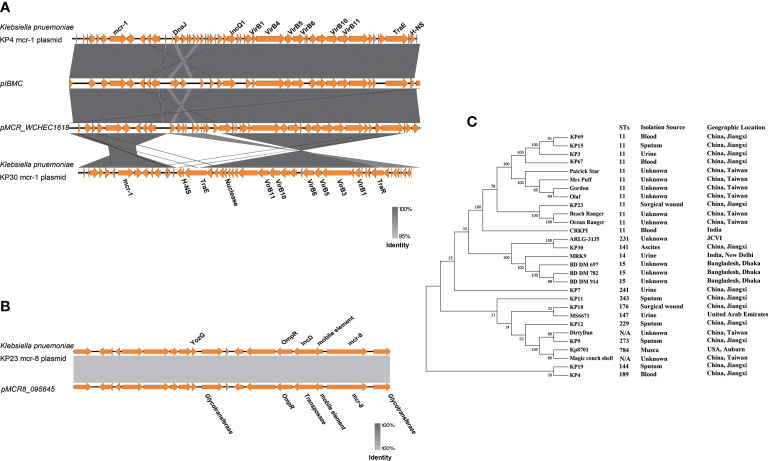
Comparative analysis of COLR-KP. **(A)** Comparative analysis of *mcr-1* related plasmids. **(B)** Comparative analysis of *mcr-8* related plasmids. **(C)** Phylogenetic analysis of core genes for COLR-KP from different area. Core genes are those genes existed in all *K. pneumoniae* genomes in this study, which were produced by a pan genome analysis using Roary pipeline (e value = 1e-5, identity = 90, coverage = 90).

Comparative plasmid analysis revealed that *mcr-1-*bearing plasmids in KP4 identified in this study had >95% nucleotide identity to the plasmid pMCR_WCHEC1618 (GenBank accession no. KY463454) isolated in Sichuan, China and plasmid pIBMC*mcr1* (GenBank accession no. MF449287) isolated in Italy. Interestingly, plasmid pMCR_KP30 displayed a huge difference from KP4, which showed large fragment inversion (**Figure 1A**). In addition, an approximately 13-kb *mcr-8*-related plasmid fragment in KP23 genome showed >99.9% identity to plasmid pMCR8_095845 (GenBank accession no. CP031883; **Figure 1B**).

Furthermore, to characterize the genetic features of the COLR-KP isolates, we performed a comparative genomic analysis. We identified 9,227 gene clusters, including 4,140 core, 2,593 cloud, and 2,494 shell genes using Roary for comparative analysis. Overall, the COLR-KP isolates were genetically diverse, suggesting that the *mcr*-bearing *K. pneumoniae* isolates were also genetically diverse, and these genes were disseminated among various *K. pneumoniae* isolates, primarily by horizontal transmission (**Figure S1**). Furthermore, we compared the colistin-resistant *K. pneumoniae* genomes from different areas. The results indicated that most of the isolates were ST11 type, and acquired colistin resistance might not have shown any strain bias (**Figure 1C**).

## Discussion

During the coronavirus disease 2019 pandemic, the problem of bacterial resistance cannot be ignored. The World Health Organization celebrated the “World Antimicrobial Awareness Week” in 2020, aiming to raise awareness regarding the problems of antimicrobial resistance and establish a united front to preserve antimicrobials and curb the development of antimicrobial resistance. Colistin is an ultimate line of refuge against lethal infections by MDR gram-negative pathogens. Unfortunately, this last-resort antibiotic has been challenged by the mobilized colistin resistance determinant *mcr* family, resulting in a rapid spread worldwide with unexpected diversity.

The overall prevalence of colistin resistance in clinical *K. pneumoniae* isolates was 4.5% (53/1,171), which is higher than that in national surveillance from the CHINET report (CHINET data, 2020. http://www.chinets.com/Data/AntibioticDrugFast). In addition, the rising prevalence trend of COLR-KP was observed with each passing year, with a significant increase from 2.8% (17/603) in 2018 to 6.3% (36/568) in 2019. However, of all patients without colistin exposure, none had a history of recent overseas travel. It is possible that the spread of colistin resistance can occur in hospitals in the absence of colistin use, which is consistent with previous study (Huang et al., 2018). The fact that the prevalence of COLR-KP increased in 2019 coincided well with the consumption of colistin in our hospital, suggesting that selective pressure from colistin use is a factor that intensifies resistance rather than a definite correlation to colistin use.

Among the COLR-KP isolates in our study, >50% were MDR, which further limited the current treatment options. Four of these isolates were KPC-producing *K. pneumoniae* ST11 clones, indicating that colistin resistance emerged among the KPC-2-producing *K. pneumoniae* pandemic ST11 clonal lineage isolates in our region. Recent clinical studies have reported that mortality rates among patients with infections caused by COLR-KPC *K. pneumoniae* isolates were higher than those among patients infected by colistin-susceptible KPC *K. pneumoniae* strains. This observation is attributable to further narrowing of available therapeutic options (Giacobbe et al., 2015).

Of particular concern were two COLR-KPC *K. pneumoniae* isolates, KP67 and KP69, which exhibited pandrug resistance (PDR) and were recovered from patients with BSI; these were resistant to all tested antimicrobials, including tigecycline. These findings suggest that colistin resistance is imparted to KPC-producing *K. pneumoniae* with minimal impact on its ability to either spread or cause invasive infection. This finding agrees with those of an earlier report (Arena et al., 2016).

The genomic features of isolates were consistent with the previously described data for other *K. pneumoniae* genomes (Wyres and Holt, 2018). MLST data showed that 13 COLR-KP isolates had diverse genetic backgrounds and five of these isolates belonged to the epidemic ST11 clone. Phylogenetic analysis also showed a genetic diversity, including those ST11 isolates. Although isolated in the same period (within a week) from the same department, the mechanisms of colistin resistance in ST11 KP67 and KP69 strains were different, indicating that the colistin resistance of these strains proceeded in a distinctly evolutionary pathway.

Of the 13 colistin-resistant KP isolates, four carried *mcr* genes, and among these, three carried *mcr-1* and one carried *mcr-8* genes. The genetic characterization of *mcr-1-*bearing plasmids showed that *mcr-1* in KP4 and KP30 was located in the transferable approximately 33-kb *IncX4* plasmid, the most prevalent version of the *mcr-1*-bearing plasmid reservoirs (Fernandes et al., 2016; Zurfluh et al., 2016). Comparative analysis revealed that the *mcr-1-*bearing plasmid pMCR_KP4 (KP4) identified in this study displayed >95% nucleotide identity to various plasmids, including pMCR_WCHEC1618 (GenBank accession no. KY463454) isolated from *E. coli* in Sichuan, located 1,490 km away from Nanchang, China, and pIBMC*mcr1* (GenBank accession no. MF449287) isolated in Italy. Interestingly, KP30 bore a different *mcr-1* plasmid, which showed a large fragment inversion. It indicated that KP30 acquires the *mcr-1* gene from a different approach. The *mcr-8.2*-bearing fragment from KP23 (pMCR_KP23), which showed a >99.9% identity to plasmid pMCR8_095845 (GenBank accession no. CP031883), was also recovered from clinical *K. pneumoniae* strain in 2016 in Sichuan, China (Ma et al., 2020).

The first identification of MCR-1-producing *K. pneumoniae* in the study hospital was in 2018, highlighting a much later emergence than that of *E. coli* in 2014 as reported previously (Quan et al., 2017). Notably, the *mcr-1*-bearing plasmid in the colistin-resistant *E. coli* was *IncX4*, which was identical to those of KP4 and KP30, implying that the *IncX4*-bearing-*mcr-1* was successfully disseminated and circulated among various species of *Enterobacteriaceae* in this hospital. Accordingly, there were questions about whether *mcr-1* was an emerging resistance trait in other countries as *mcr-1*-positive isolates were collected as early as in 2005 in France (Haenni et al., 2016).

Of a particular concern is the transfer of a plasmid with *mcr-1* into carbapenemase-producing isolates intra-hospital, which leads to nosocomial infections with PDR strains, leaving no beneficial treatment options available. In our study, we did not detect *mcr-1* gene in all KPC-2-producing COLR-CRKP isolates. Nevertheless, we detected two PDR strains resistant to all antibiotics, including tigecycline, with the colistin resistance associated with *mgrB* inactivation. These patients had no colistin exposure history before the specimen collection; consequently, tracing the origin of selective pressure for these isolates was difficult.

Plasmid pMCR_KP4 and pMCR_KP30 successfully transferred the *mcr-1* gene to *E. coli* J53AZ^R^, which harbored an intact set of conjugative transfer genes to facilitate the transfer of plasmids among various isolates, consistent with its ability to conjugate into *E. coli* J53AZ^R^, as described above. In contrast, the *mcr-8.2* could not be transferred from the KP23 isolate to the recipient strain by conjugation despite several attempts. Moreover, no genes encoding conjugation-related proteins were found in the sequence of pMCR_KP23; therefore, it is suggested that the *mcr-8.2-*bearing plasmid in this strain was non-self-transmissible, which is consistent with the results of a previous report (Ma et al., 2020). Although the IS*3*-like family was detected in this plasmid, it could mobilize the intervening genetic components of *mcr-8.2* or acquire colistin resistance.

Interestingly, KP18 was an *mcr-1*-ESBL-producing isolate, which also carried a novel plasmid-borne RND-type tigecycline-resistance determinant *tmexCD1*-*toprJ1* (Lv et al., 2020). More recently, a research group first identified the *tmexCD1*-*toprJ1* located on the conjugative *IncFIA* plasmid (pHNAH8I-1) in a PDR *K. pneumoniae* isolate obtained from a chicken in Sichuan. This strain also carried both carbapenem-resistance gene *bla*_NDM-1_ and colistin- resistance genes *mcr-1* and *mcr-8* (Lv et al., 2020). Although *tmexCD1-toprJ1* mainly spreads among *K. pneumoniae* isolates from food animals, the simultaneous appearance of mobilized colistin- and tigecycline-resistance genes in clinical isolates is an alarming evolutionary trend, which may lead to rapid horizontal transfer in hospital settings; this requires attention for a continuous and meticulous surveillance.

In the present study, the most common chromosomal mutation associated with colistin resistance was the T246A amino acid substitution in *pmrB*. This substitution was identified in most COLR-KP isolates and reported in colistin-resistant *K. pneumoniae* in a Tunisian Teaching Hospital (Jaidane et al., 2018). However, the T246A was predicted as neutral mutation that was most likely unrelated to colistin resistance, consistent with of the findings of a previous report (Longo et al., 2019). All ST11 isolates had R256G and T246A amino acid substitutions in *pmrB*, similar to that previously described in ST258 isolates (Aires et al., 2016), and the ST11 is a close variant of the international pandemic clone ST258. Furthermore, some isolates presented several new mutations as amino acid substitutions: S203P, T240M, and V257A in *PmrB*; E35A and M66I in *PmrA*; and V24G in *PhoQ*. These mutations were predicted to be neutral, with no effect on the biological functions of these proteins. The underlying mechanisms of colistin resistance in GNB are complex and not completely understood yet (El-Sayed Ahmed et al., 2020). According to our results, there still exists some unknown molecular mechanism leading to colistin resistance, which requires further investigations. However, this prediction is not definitive, and further investigation is needed to confirm the relationship between these mutations and colistin resistance.

MgrB mutation was the most frequently reported molecular mechanism of colistin resistance in *K. pneumoniae* (Cannatelli et al., 2014), whereas only three isolates possessed *mgrB* mutations in our study. The ST105 KP12 and ST11 KP69 isolates had complete deletion; the ST11 KP67 isolate had a truncated gene due to a promoter deletion. Insertional inactivation caused by diverse ISs at different locations within the *mgrB* gene was often responsible for acquired colistin resistance in *K. pneumoniae* (Nordmann et al., 2016). Some complete deletions of the *mgrB* locus had been reported as well. Of note, isolate KP67 was significantly more virulent and exhibited resistance to tigecycline and carbapenem, as well as to colistin, causing BSI.

Various resistance genes were carried by these COLR-KP isolates, which conferred resistance to carbapenems, cephalosporins, fluoroquinolones, aminoglycosides, macrolides, sulfonamides, tetracyclines, and trimethoprims. Four carbapenem-resistant COLR-KP isolates produced KPC-2 as the predominant carbapenemase in the world and China (Munoz-Price et al., 2013; Zong et al., 2020). In addition, critical virulence factors associated with the hypervirulent *K. pneumoniae*, including aerobactin (*iucABCD* and *iutA*), salmochelin (*iron* and *iroBCD*), and yersiniabactin (*ybt*), were found in the isolates KP4, KP11, KP19, and KP67. The combination of hypervirulence and high drug resistance also resulted in veritable “superbug,” thus making infection control intractable.

## Conclusion

COLR-KP emerged in our hospital and was increasing with each passing year. Although the molecular determinants of colistin resistance in *K. pneumoniae* clinical isolates in our region are still chromosomal mutations, *mcr-1* and *mcr-8* have been detected in MDR *K. pneumoniae* isolates without colistin use. Moreover, the coexistence of plasmid-borne *mcr-1* and *tmexCD1-toprJ1* in *K. pneumoniae* further threatens public health. Of a particular concern is the simultaneous emergence of hypervirulence and colistin–tigecycline–carbapenem resistance in an epidemic clone ST11 *K. pneumoniae* isolate, causing a lethal and untreatable infection.

## Data Availability Statement

The data presented in the study are deposited in the NCBI repository, accession number PRJNA718703 can be found below:https://www.ncbi.nlm.nih.gov/bioproject/?term=PRJNA718703.

## Ethics Statement

The studies involving human participants were reviewed and approved by Ethics Committee of the First Affiliated Hospital of Nanchang University. The patients/participants provided their written informed consent to participate in this study.

## Author Contributions

XH, JG, and LZ designed the study. YLL, YQL, and XL wrote the manuscript. LZ reviewed the manuscript. NH and QL managed the strains and data collection. ZW, WZ, XL, and YQL prepared the figures and tables. All authors contributed to the article and approved the submitted version.

## Funding

This work was supported by the National Natural Science Foundation of China (32060040 and 31760261), the Natural Science Foundation of Jiangxi Province (20202BABL216084, 20192ACBL21042, 20202BAB216045, 20192BBG, and 20204BCJL23054), the Science and Technology Research Project of Education Department of Jiangxi Province (GJJ180130), and the Major Science and Technology Project of Jiangxi Province (20181BBG70030).

## Conflict of Interest

The authors declare that the research was conducted in the absence of any commercial or financial relationships that could be construed as a potential conflict of interest.

The reviewer QL declared a past co-authorship with several of the authors, LZ and YZ, to the handling editor.

## Publisher’s Note

All claims expressed in this article are solely those of the authors and do not necessarily represent those of their affiliated organizations, or those of the publisher, the editors and the reviewers. Any product that may be evaluated in this article, or claim that may be made by its manufacturer, is not guaranteed or endorsed by the publisher.

## Supplementary Material

Supplementary Figure 1 Comparative genomics of 13 COLR-KP isolated in this study. **(A)** Pan genomic analysis of 13 COLR-KP isolates. **(B)** Phylogenetic analysis of 13 COLR-KP genomes using their core proteins. Gene matrix showed genes present (blue) or absent (blank) in the *K. pneumoniae* genomes.
