# Supplementary figures and images for: Molecular Mechanisms of Colistin Resistance in Klebsiella pneumoniae in a Tertiary Care Teaching Hospital
Source: Front Cell Infect Microbiol. 2021 Oct 26;11:673503. doi: 10.3389/fcimb.2021.673503 (PMC8576191; doi:10.3389/fcimb.2021.673503)

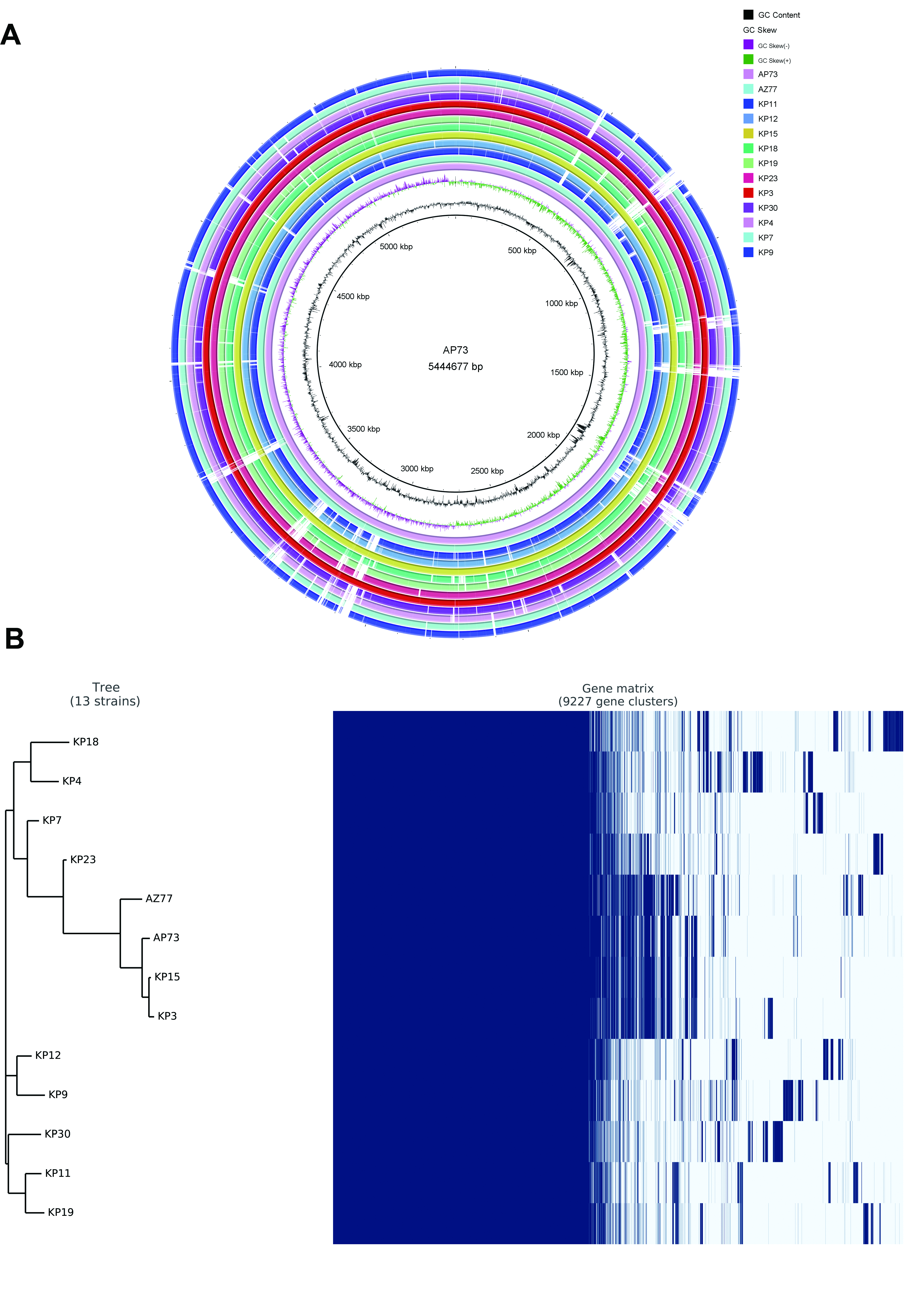

Supplement: Supplementary Figure 1 — Comparative genomics of 13 COLR-KP isolated in this study. (A) Pan genomic analysis of 13 COLR-KP isolates. (B) Phylogenetic analysis of 13 COLR-KP genomes using their core proteins. Gene matrix showed genes present (blue) or absent (blank) in the K. pneumoniae genomes. [file Image_1.tif]
