# Supplementary material for: Molecular Mechanisms of Colistin Resistance in Klebsiella pneumoniae in a Tertiary Care Teaching Hospital
Source: Front Cell Infect Microbiol. 2021 Oct 26;11:673503. doi: 10.3389/fcimb.2021.673503 (PMC8576191; doi:10.3389/fcimb.2021.673503)
Supplement: Supplementary file 2 [file Table_1.docx]

**Table S1 Primers used for detection mcr genes in this study**

| Prime name | Sequence (5'-3') | size(bp) | Reference |
| --- | --- | --- | --- |
| mcr-1_fw | AGTCCGTTTGTTCTTGTGGC | 320 | Rebelo et al., 2017 |
| mcr-1_rev | AGATCCTTGGTCTCGGCTTG |  |  |
| mcr-2-fw | CAAGTGTGTTGGTCGCAGTT | 715 | Rebelo et al., 2017 |
| mcr-2-rev | TCTAGCCCGACAAGCATACC |  |  |
| mcr-3-fw | AAATAAAAATTGTTCCGCTTATG | 929 | Rebelo et al., 2017 |
| mcr-3-rev | AATGGAGATCCCCGTTTTT |  |  |
| mcr-4_fw | TCACTTTCATCACTGCGTTG | 1116 | Rebelo et al., 2017 |
| mcr-4_rev | TTGGTCCATGACTACCAATG |  |  |
| mcr-5_fw | ATGCGGTTGTCTGCATTTATC | 1644 | Rebelo et al., 2017 |
| mcr-5_rev | TCATTGTGGTTGTCCTTTTCTG |  |  |
| mcr-6_fw | GTCCGGTCAATCCCTATCTGT | 556 | Borowiak et al., 2020 |
| mcr-6_rev | ATCACGGGATTGACATAGCTAC |  |  |
| mcr-7_fw | TGCTCAAGCCCTTCTTTTCGT | 892 | Yang et al., 2018 |
| mcr-7_rev | TTCATCTGCGCCACCTCGT |  |  |
| mcr-8_fw | AACCGCCAGAGCACAGAATT | 667 | Wang et al., 2018 |
| mcr-8_rev | TTCCCCCAGCGATTCTCCAT |  |  |
