# Supplementary material for: Molecular Mechanisms of Colistin Resistance in Klebsiella pneumoniae in a Tertiary Care Teaching Hospital
Source: Front Cell Infect Microbiol. 2021 Oct 26;11:673503. doi: 10.3389/fcimb.2021.673503 (PMC8576191; doi:10.3389/fcimb.2021.673503)
Supplement: Supplementary file 3 [file Table_2.docx]

**Table S2 General features of colistin-resistant *Klebsiella pneumoniae* genomes**

| Isolates | Genome features | | | | | | | |
| --- | --- | --- | --- | --- | --- | --- | --- | --- |
|  | Genome length (bp) | Contigs | No. CDS | G+C content (%) | Proteins  with functional assignment | Hypothetical protiens | rRNA | tRNA |
| KP3 | 5,867,990 | 322 | 6011 | 57.0 | 5113 | 898 | 13 | 81 |
| KP4 | 5,743,947 | 189 | 5791 | 56.9 | 3210 | 928 | 18 | 83 |
| KP7 | 5,381,464 | 241 | 3521 | 57.5 | 4655 | 445 | 10 | 56 |
| KP9 | 5,499,066 | 273 | 5514 | 57.3 | 4789 | 468 | 17 | 89 |
| KP11 | 5,284,652 | 243 | 5257 | 57.5 | 4591 | 666 | 14 | 88 |
| KP12 | 5,456,624 | 229 | 5407 | 57.2 | 3106 | 708 | 15 | 84 |
| KP15 | 5,799,117 | 302 | 5929 | 57.1 | 3331 | 887 | 13 | 81 |
| KP18 | 5,759,268 | 176 | 5768 | 56.7 | 3168 | 658 | 19 | 90 |
| KP19 | 5,403,968 | 144 | 5283 | 57.3 | 3083 | 644 | 12 | 83 |
| KP23 | 5,474,432 | 280 | 5502 | 57.3 | 4730 | 772 | 14 | 85 |
| KP30 | 5,429,479 | 141 | 3548 | 57.3 | 3089 | 715 | 8 | 52 |
| KP67 | 5,826,170 | 4(final) | 6100 | 56.9 | 5159 | 941 | 25 | 85 |
| KP69 | 5,958,900 | 6(final) | 5973 | 57.1 | 5132 | 841 | 27 | 87 |
